# categoryCompare: high-throughput data meta-analysis using gene annotations

**DOI:** 10.1186/1471-2105-12-S7-A16

**Published:** 2011-08-05

**Authors:** Robert M Flight, Jeffrey C Petruska, Benjamin J Harrison, Eric C Rouchka

**Affiliations:** 1 Department of Anatomical Sciences and Neurobiology, University of Louisville, Louisville, KY, 40292, USA; 2 Department of Computer Engineering and Computer Science, University of Louisville, Louisville, KY, 40292, USA

## Background

**Figure 1:**
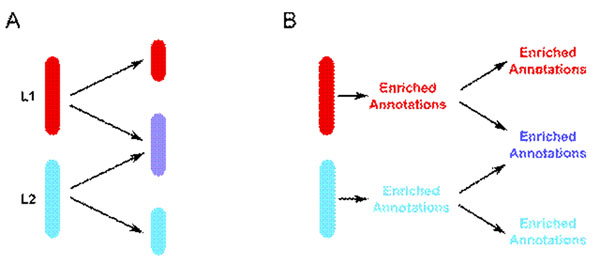
**A** - Usual method of high-throughput experiment meta-analysis comparing gene lists (L1 and L2) directly. **B** - categoryCompare compares the gene lists on the basis of enriched annotations.

Many current DNA microarray and other high-throughput data meta-analysis studies concentrate on deriving a concordant list of genes across many experiments to discover the "true" genes responsible for a particular disease process or biological pathway or cellular response (Figure 1A). However, by concentrating on the genes in common, similarities or differences that exist at a pathway or process level may be missed.

## Results

We describe a meta-analysis approach that allows comparison and contrast of gene lists at the level of categorical annotation (pathway or Gene Ontology annotations). This categorical evaluation compares enriched annotations between gene lists (Figure 1B), and displays the results graphically to allow intuitive visualization and exploration of the similarities and differences. False discovery correction via simulation is implemented to control for the effect of different sized gene lists as inputs.

## Conclusions

The approach was tested using two gene lists, genes involved in the response to denervation in muscle (a literature compendium), and in skin (experimentally determined). Using the categorical comparison highlights known biological processes that are common in the two cases, while also allowing one to easily see areas of difference that are not apparent from examining the gene lists alone.

## Availability

categoryCompare is available as a Bioconductor package, and a web interface (using RApache) has also been developed to facilitate use in the wider research community.

